# Diamond knife dissection technique for nerve preservation during resection of large vestibular schwannomas

**DOI:** 10.3171/2021.7.FOCVID21104

**Published:** 2021-10-01

**Authors:** Julia Shawarba, Cand Med, Matthias Tomschik, Karl Roessler

**Affiliations:** Department of Neurosurgery, Medical University/AKH, Vienna, Austria

**Keywords:** vestibular schwannoma, hearing preservation, facial nerve preservation, diamond knife dissection

## Abstract

Facial and cochlear nerve preservation in large vestibular schwannomas is a major challenge. Bimanual pincers or plate-knife dissection techniques have been described as crucial for nerve preservation. The authors demonstrate a recently applied diamond knife dissection technique to peel the nerves from the tumor capsule. This technique minimizes the nerve trauma significantly, and complete resection of a large vestibular schwannoma without any facial nerve palsy and hearing preservation is possible. The authors illustrate this technique during surgery of a 2.6-cm vestibular schwannoma in a 27-year-old male patient resulting in normal facial function and preserved hearing postoperatively.

The video can be found here: https://stream.cadmore.media/r10.3171/2021.7.FOCVID21104

## Transcript

This is Dr. Karl Roessler and herein I will demonstrate a microsurgical resection of a 2.6-cm vestibular schwannoma using a diamond knife for the dissection with the goal of preserving facial nerve function and hearing. To the best of our knowledge, this is the first surgical video to demonstrate the use of this technique for vestibular schwannoma resection.^1,2^

### 0:50 Patient History.

The patient is a 27-year-old male who presented with left-sided hearing loss, tinnitus, loss of balance, and dizziness. Neurological exam was nonfocal without any additional neurological deficits. MRI scan showed a classic “ice cream on cone” vestibular schwannoma, measuring 26 mm in diameter in the left cerebellopontine angle (CPA) extending into the left internal acoustic meatus.

### 1:37 Patient Positioning.

The patient was positioned semisetting, with concurrent intraoperative monitoring using continuous facial nerve electromyography and brainstem auditory evoked potentials.^3^ Additionally, the patient was continuously monitored with intraoperative transesophageal echocardiography to detect and prevent any venous air embolism.^4^

### 1:57 Tumor Debulking.

After retrosigmoidal craniectomy and dura opening, the cerebellum was gently retracted and the CPA was exposed. Then drilling of posterior wall of the IAC was performed using a 2-mm diamond drill. Initially, the tumor was debulked using the ultrasonic surgical aspirator (CUSA).

### 2:23 Intrameatal Tumor Resection.

The facial nerve is identified by stimulation.^5^ Consequently, the intrameatal tumor portion was enucleated by using a sharp, round plate-knife. After that, a diamond knife was inserted to detach the cochlear nerve from the tumor capsule. Again, the facial nerve is identified on the brainstem by electrophysiological stimulation. After identifying the facial nerve by electric stimulation, the nerve was gently dissected from the tumor by dividing the arachnoidal membrane between the nerve and the tumor capsule using the diamond knife.

### 3:32 Diamond Knife Dissection Technique.

This worked perfectly well by using a sharp diamond knife for the dissection, which precisely allowed the dissection of the nerve fibers and minimized damaging the nerve. With the same technique, it was possible to complete the dissection of the tumor on the meatal part of the facial nerve, avoiding any hazardous maneuver to the nerve. [Continuing with the blunt dissection of the lateral circumference of the lesion following the facial nerve’s extent, adopting the same technique of dissection.]

### 5:12 Tumor Removal Completed.

Finally, the remaining part of the tumor was detached by using the microscissors. The cochlear nerve was inspected and covered with Surgicel. The completely tumor-free facial nerve is covered with calcium antagonist–soaked Gelfoam.

### 6:12 Closure.

After tumor removal, a free muscle graft and fibrin sealant were applied to occlude any opened air cells at the internal auditory canal and to prevent risk of postoperative CSF leakage. Dura closure was then performed in a multilayer, watertight fashion. A PMMA (polymethylmethacrylate) cranioplasty was used to replace the bone and held with screwed titanium plates following layer-by-layer closure of the muscle and skin.

### 6:52 Postoperative Course.

The extubation was performed immediately after the surgery, and the patient woke up without any neurological deficits despite reduced hearing on the tumor site. Postoperative audiogram below shows useful hearing preserved. This is his postoperative MRI, which demonstrates complete tumor resection. Postoperatively, the patient was neurologically intact, had no facial palsy and still useful hearing on his left ear, and was discharged at home after 1 week.

## Disclosures

The authors report no conflict of interest concerning the materials or methods used in this study or the findings specified in this publication.

## Author Contributions

Primary surgeon: Roessler. Assistant surgeon: Shawarba, Tomschik. Editing and drafting the video and abstract: Roessler, Shawarba. Critically revising the work: Shawarba, Tomschik. Reviewed submitted version of the work: all authors. Approved the final version of the work on behalf of all authors: Roessler. Supervision: Roessler.
